# Laboratory Investigation of Nanofluid-Assisted Polymer Flooding in Carbonate Reservoirs

**DOI:** 10.3390/nano12234258

**Published:** 2022-11-30

**Authors:** Kassymzhomart Ulasbek, Muhammad Rehan Hashmet, Peyman Pourafshary, Rizwan Muneer

**Affiliations:** 1 School of Mining and Geosciences, Nazarbayev University, Astana 010000, Kazakhstan; 2 Department of Chemical and Petroleum Engineering, United Arab Emirates University, Al-Ain 15551, United Arab Emirates

**Keywords:** EOR, nanoparticles, polymer, wettability alteration, polymer viscosity, polymer retention, oil recovery

## Abstract

In the petroleum industry, the remaining oil is often extracted using conventional chemical enhanced oil recovery (EOR) techniques, such as polymer flooding. Nanoparticles have also greatly aided EOR, with benefits like wettability alteration and improvements in fluid properties that lead to better oil mobility. However, silica nanoparticles combined with polymers like hydrolyzed polyacrylamide (HPAM) improve polymer flooding performance with better mobility control. The oil displacement and the interaction between the rock and polymer solution are both influenced by this hybrid approach. In this study, we investigated the effectiveness of the injection of nanofluid-polymer as an EOR approach. It has been observed that nanoparticles can change rock wettability, increase polymer viscosity, and decrease polymer retention in carbonate rock. The optimum concentrations for hydrolyzed polyacrylamide (2000 ppm) and 0.1 wt% (1000 ppm) silica nanoparticles were determined through rheology experiments and contact angle measurements. The results of the contact angle measurements revealed that 0.1 wt% silica nanofluid alters the contact angle by 45.6°. The nano-silica/polymer solution resulted in a higher viscosity than the pure polymer solution as measured by rheology experiments. A series of flooding experiments were conducted on oil-wet carbonate core samples in tertiary recovery mode. The maximum incremental oil recovery of 26.88% was obtained by injecting silica nanofluid followed by a nanofluid-assisted polymer solution as an EOR technique. The application of this research will provide new opportunities for hybrid EOR techniques in maximizing oil production from depleted high-temperature and high-salinity carbonate reservoirs.

## 1. Introduction

Most mature oil reservoirs experience a gradual decline in oil production over time and discovering new oil resources alone would not be enough in the future to meet the rising global demand for energy [1]. As a result, the oil industry is focusing on enhanced oil recovery (EOR) techniques to recover the maximum remaining trapped oil [2]. Hence, it is crucial to develop innovative, cutting-edge EOR techniques to maximize crude oil production. Understanding rock and fluid properties can lead to the development of new techniques for extracting trapped oil from hydrocarbon reservoirs [3].

When the water flooding approach is ineffective due to the viscous fingering phenomenon that causes early water breakthrough, polymer flooding may be utilized for better mobility control [4]. To improve the viscosity of the injection fluid, high molecular weight water-soluble polymers are mixed with the water phase [5,6]. The high viscosity of the injection fluid improves conformance control and slug mobility while also eradicating the problem of viscous fingering. As a result, early water breakthrough, which is common in the waterflooding method, is reduced, and incremental oil recovery is obtained [7,8,9]. The majority of big oil fields, including the Daqing oilfield in China and the East Bodo reservoir in Canada, have been using polymer flooding for decades. Polymer flooding has also been successfully used at the Tambaredjo field in Suriname, the Marmul field in Oman, and the Pelican Lake field in Canada. With these success stories, polymer flooding has maintained its expanding significance in the contemporary energy market [10].

Processes in the petroleum industry, such as exploration, fracturing, enhanced oil recovery (EOR), fines migration control, and refining, have been transformed by the use of nanoparticles on both upstream and downstream levels [11,12,13,14,15]. Nanoparticles are widely used in EOR, which results in a higher volume of oil produced during extraction and a quicker return on investment [16]. Ogolo et al. (2012) published results from EOR studies using various nanoparticles such as magnesium oxide, aluminum oxide, zirconium oxide, zinc oxide, iron oxide, tin oxide, nickel oxide, hydrophobic silicon dioxide, and silicon dioxide treated with silane, which showed increased recovery and hydrocarbon output. Changes in rock wettability, polymer viscosity, interfacial tension, and mobility control are among the benefits of utilizing nanoparticles [17,18].

Wettability alteration is considered to be one of the key mechanisms in the EOR techniques [19]. By modifying the rock wettability from an oil-wet to a highly water-wet system, nanoparticles played a significantly vital role in the production of hydrocarbons [20]. Understanding how the wettability of the reservoir shifts from oil-wet to water-wet conditions during the oil recovery process depends on the oil disjoining pressure. Recently, researchers have investigated the application of nanoparticles in wettability modification for enhanced oil recovery [21,22,23,24,25,26]. The potential of nanoparticles to alter the wettability of the rock to a water-wet state and increase the recovery factor greatly improves when they work in conjunction with other additives such as polymers and surfactants. Chunbao et al. (2022) recently used a novel approach to boost oil recovery by employing quantum dots-stabilized foam fluid. They achieved the greatest ever documented incremental oil recovery of 18.2% using nanoparticle-stabilized foam fluids [27]. Bila et al. (2019) investigated how polymer-coated silica nano-fluid affected oil recovery in a highly oil-wet environment and observed that the rock’s wettability was changed to water-wet, leading to a higher oil recovery [28]. The results also matched the experimental findings of Gbadamosi [29].

The petroleum industry has become interested in novel polymer-coated nanoparticles due to their better solubility and durability, increased emulsion stabilization, and improved mobility in porous media. Several studies have highlighted these features of nanoparticles to increase oil recovery [29,30]. Rodriguez et al. (2009) and Zhang et al. (2015) discovered that silica nanoparticles and polymer mixtures have a great transport behavior across rock pores of different sizes with weak retention because of their reversible adsorption on the solid surface [31,32]. Ponnapati et al. (2011) observed that polymer-modified silica nanoparticles have the ability to displace residual oil, and in a laboratory trial, they produced 7.9% of the OOIP [33].

Behzadi and Mohammadi (2016) claimed that polymer/silica nanoparticle solutions may control the interfacial tension (IFT) between oil and water and change the wettability of glass micromodel from oil-wet to water-wet, producing a better EOR effect than unmodified silica nanoparticles [34]. In the work by Saha et al. (2018), core flooding studies using silica nanoparticle-assisted polymer flooding were undertaken, and the IFT was lowered, resulting in more consistent oil-water emulsions. Furthermore, the wettability of the system also changed from an intermediate oil-wet system to a strongly water-wet system [35]. Maghzi et al. (2013) investigated the effect of silica nanoparticle dispersion on polymer flooding considering various fluid salinities [36]. Luo et al., (2006) investigated that polymer degradation can be greatly reduced by adding silica nanoparticles to the polymer solution [37]. The findings from Bila et al. (2019) core flooding studies show that silica nanoparticles and a polymer mixture increased the oil recovery factor from 2.6% to 5.2% in tertiary recovery mode [28]. The current research provides new insight and demonstrates the effectiveness of nanofluid-assisted polymer flooding in high-salinity and high-temperature carbonate reservoirs. In addition, the oil displacement process is investigated using different injection scenarios to optimize the process. Polymers are known to promote bridging-induced flocculation of nanoparticles in water, which improves their rheological properties [38,39]. The interaction of particles and polymers may also increase the tolerance of polymer solutions to salinity and high temperatures [40,41]. Maurya et al. (2016) reported that at higher concentrations of silica particles, when practically all of the polymeric chains are adsorbed on the silica surface, the influence of salinity on viscosity is relatively negligible, and the viscosity of the silica/PAM combination is substantially higher than that of the PAM solution. The use of polymer/silica nanoparticles as an EOR approach offers various benefits, including increased viscosity, reduced disjoining pressure, wettability alteration, and reduced IFT [42].

## 2. Materials and Methods

During the first phase, silica nanofluids and modified synthetic polymer solutions were prepared and used in standalone and hybrid EOR designs to analyze the improvement in oil recovery. Zeta potentials were measured to check the stability of the prepared nanofluids. Contact angle and rheology measurements were used to determine the optimum concentrations of silica nanoparticles and modified synthetic polymer for high temperature and high salinity conditions. The next phase involved looking into how the hybrid application of chemicals altered the rheological properties of injection fluids. Various designed flooding sequences were employed using polymer, nanofluid, and nanofluid-assisted polymer solutions. The most effective nanofluid-assisted polymer solution was identified based on fluid/rock interactions and oil displacement results.

### 2.1. Core Samples

**Figure 1:**
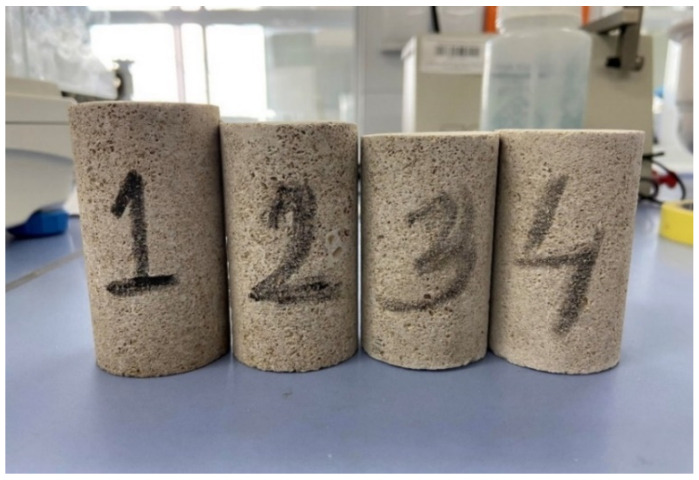
Indiana limestone core samples used in coreflood-1 through 4.

In this research, Indiana limestone rock samples were used, as seen in Figure 1. A long core was cut into four small core samples for core flooding experiments and twelve pellets for contact angle measurements. The pellets have a 1.5-inch diameter. Table 1 lists the key parameters for core samples.

After cutting the core samples and measuring their dry weights, lengths, and diameters, they were saturated with formation water using a core saturator to create initial reservoir conditions. To quantify permeabilities and irreducible water saturations, cores were first flooded with formation water and then with oil, as reported in Table 2. The core samples were then aged for six months at 80 °C in an oven to make them completely oil-wet.

### 2.2. Crude Oil

In this research, crude oil with a 35 API gravity was used, which was taken from a field in the Caspian Sea in Kazakhstan. The oil was filtered to remove contaminants like water, gas, and solid particles. The oil properties are presented in Table 3.

### 2.3. Brines

40,000 ppm of brine was chosen as an injection fluid, while 183,000 ppm of brine was employed as formation water for this research. The highest salinity at which silica nanofluid remains stable is 40,000 ppm for brine, according to Zhangaliyev et al. (2022) [43]. In this study, formation water with a salinity of 182,980 ppm was used, which is a typical concentration of formation water for carbonate fields in Kazakhstan. The core samples were saturated with formation water to mimic the initial reservoir conditions. The ionic composition of the formation water and the injection brine are given in Table 4.

### 2.4. Polymer

A commercial synthetic polymer called SUPERPUSHER SAV 10 was utilized. It is a partially hydrolyzed polyacrylamide (HPAM) polymer supplied by SNF Floerger in a white powder. The polymer used had a molecular weight of 2–4 million g/mol (Daltons). Chemically, this polymer can withstand a high temperature of 80 °C and a salinity of 183,000 ppm [43,44].

### 2.5. Silica Nanoparticles

In this research, silicon dioxide (SiO_2_) nanoparticles from SkySpring Nanomaterials (Houston, TX, USA) were utilized. The silica nanoparticles used in the EOR process are the most efficient [23,28,45]. Table 5 provides the key characteristics of the nanoparticles.

### 2.6. Fluid Preparation

Formation water and injection brine were prepared by adding calculated amounts of salts, such as NaCl, CaCl_2_·2H_2_O, and MgCl_2_·6H_2_O to the distilled water. To avoid the development of “fish-eyes”, the dry polymer was uniformly added to distilled water while being stirred with a magnetic stirrer at a speed of 600 rpm. After adding the polymer, the solution was stirred for three hours with a magnetic stirrer set to 150 rpm to prevent mechanical degradation. For nanofluid preparation, the silica nanoparticles were dispersed and homogenized using an ultrasonic homogenizer at 70 °C for 45 min. These parameters were determined experimentally in our previous study to obtain a homogeneous and stable nanofluid [43]. Both the polymer solution and the nanofluid were prepared in 40,000 ppm salinity. After one hour of cooling, the polymer was added to the nanofluid and mixed with a magnetic stirrer at a slow speed. To ensure a salinity of 40,000 ppm in the nanofluid-polymer solution, all calculations were carefully performed.

### 2.7. Zeta Potential Measurements

Zeta potential describes the electrostatic interaction between cells and particles in a fluid environment. The ionic envelope that surrounds a particle typically has two layers; the stern layer, which tightly holds the ions, and the diffuse layer, which contains loose ions. The combined effect of these two electrostatic layers is known as the electric double layer (EDL). Zeta potential refers to the potential difference between these two layers. To ascertain the stability of nanofluids at various concentrations of 0.05, 0.1, and 0.15 wt% (500, 1000, and 1500 ppm) and different salinities, several zeta potential tests were carried out. Generally, stable particles have zeta potentials greater than or equal to +30 mV or less than −30 mV. The Malvern Zetasizer Nano ZS (Malvern Panalytical Ltd., Grovewood Road, Malvern, UK) was used to measure the zeta potential of nanofluids both before and after salt addition. Each test was performed three times to get a high-quality measurement. The optimum concentration of nanoparticles was determined when the nanofluid was stable for a longer time.

### 2.8. Contact Angle Measurements

To determine the best concentration of silica nanoparticles to change rock wettability, contact angle measurements were carried out. The limestone pellets were dried in an oven for 24 h. A vacuum pump with 74 mPa pressure attached to a desiccator was used to remove the air after the light oil was introduced to the pellets. The saturated oil pellets were then aged for a further two months at 80 °C in the same light crude oil. The OCA 15EC apparatus (DataPhysics Instruments, 70794 Filderstadt, Germany) measured the initial wettability of the core using the captive bubble method at room temperature, where the dropping phase was light crude oil, and the ambient phase was formation water. The limestone pellets were then submerged in silica-based nanofluids for 48 h after the initial contact angle was measured. To check whether the wettability was altered by nanofluids, the contact angles were measured again. For each test, three measurements were taken, and the average value was reported. Less than 75° contact angle was reported as a water-wet case, between 75° and 115° as an intermediate wet case, and more than 115° as an oil-wet case [46].

### 2.9. Rheology Measurements

The effect of the presence of nanoparticles, temperature, salinity, and chemical concentrations was studied by evaluating the rheological behavior of polymer and nanofluid-assisted polymer solutions. The test was performed on the MCR 301 device (Anton Paar Graz, Austria) with a cylindrical cup system to take measurements at high temperature. With varying concentrations of the SAV 10 polymer (1000, 1500, 2000, and 2500 ppm) and the optimum concentration of silica nanoparticles determined from contact angle measurements, all experiments were conducted at ambient temperature and up to 80 °C with a brine salinity of 40,000 ppm. The viscosity was measured and compared at a shear rate of 10 s^−1^ at 80 °C to determine the optimum condition. Additionally, the rheological behavior of nanofluid-assisted polymer solutions was also investigated and compared with the polymer solutions at different concentrations.

### 2.10. Coreflooding Experiments

**Figure 2:**
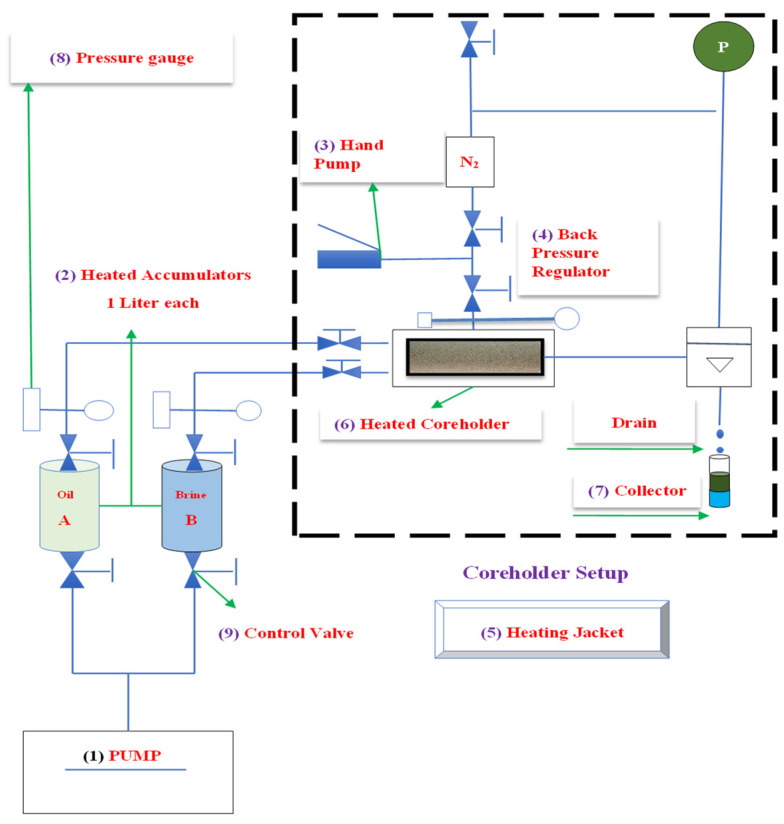
Schematic of the core flooding equipment [47].

Four core flooding experiments were carried out to assess the efficacy of polymer, nanofluids, and hybrid nano-assisted polymer on oil displacement. The details of injection scenarios used in core floods are shown in Table 6. The setup of the core flooding apparatus is illustrated in Figure 2.

## 3. Results and Discussion

### 3.1. Zeta Potential Results

Table 7 presents the results of the zeta potentials as a function of silica nanoparticle concentration before and after the addition of salts. All zeta potential tests were carried out at an ambient temperature of 25 °C. Silica nanofluid of 0.1 wt% concentration was highly stable before salt was added. However, it was observed that a high salinity condition reduced nanofluid stability. A reduction in nanofluid stability was attributed to the compaction of EDL in high salinity conditions and the dominance of high attraction force between silica nanoparticles, resulting in agglomeration. The zeta potential of the silica nanofluid at 0.1 wt% showed −6.3 mV and was the most stable at a high salinity environment of 40,000 ppm; therefore, this concentration was selected for this study.

### 3.2. Optimization of Nanofluid and Polymer Concentration

**Figure 3:**
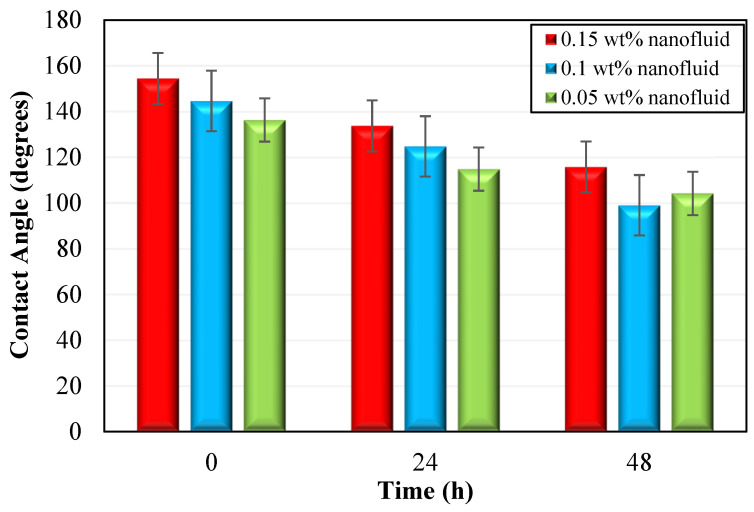
Contact angles for various nanofluid concentrations (0.05, 0.1, 0.15 wt%).

**Figure 4:**
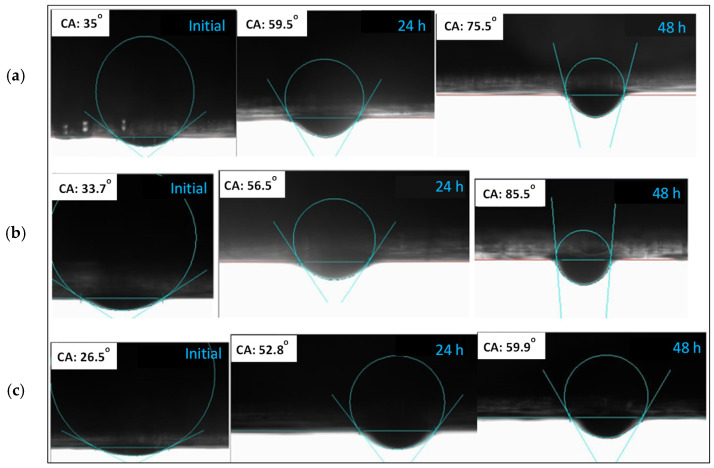
Contact angle measurements from OCA 15EC (**a**) 0.05 wt% (**b**) 0.1 wt% (**c**) 0.15 wt%.

Figure 3 illustrates the measurement of wettability alteration employing nanofluids at 0.05, 0.1, and 0.15 wt% (500, 1000, and 1500 ppm) concentrations with 40,000 ppm salinity. We performed three sets of experiments to measure contact angles, and the average angle was used in the analysis. Figure 4 shows only one example of the change in contact angles caused by silica nanofluids at various concentrations. The contact angles were measured using oil-aged carbonate pellets. On the left side of Figure 4, it is seen that the pellets were oil-wet where oil spread to the surface of carbonate rock. The ambient phase was formation water, while the dropping phase was light crude oil. As a result, it indicates that silica nanofluid has the ability to change the wettability of carbonate rock towards a water-wet state. It should be noted that in Figure 4, contact angles are subtracted from 180°. For silica nanofluid concentrations of 0.05 and 0.15 wt%, the corresponding change in contact angle from an oil-wet state to a water-wet state was 32.13° and 38.74°. However, the maximum change in the contact angle was caused by 0.1 wt% silica nanofluid which was 45.6°. Figure 3 was used to calculate the corresponding change in contact angles for 0, 24, and 48 h for each nanofluid.

The modified synthetic polymer SAV 10 was tested at ambient temperature (25 °C) and high temperature (80 °C). The key parameter influencing viscosity is temperature, which has an impact on the polymer’s stability and viscosity. As temperature increases, the viscosity of a polymer solution decreases due to thermal degradation. For this research, the target viscosity for the polymer solution was set to be 3–4 cP at 80 °C for a shear rate of 10 1/s.

**Figure 5:**
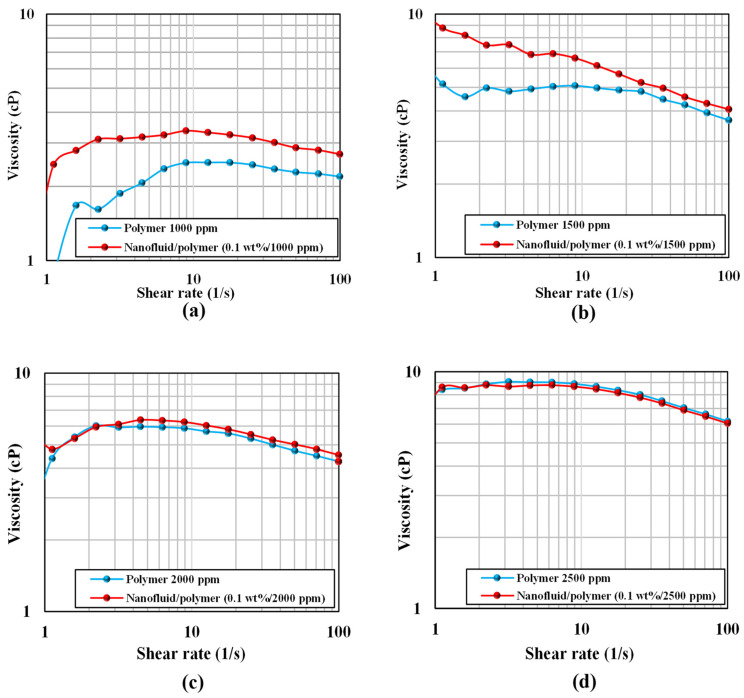
Comparison of pure polymer and nanofluid-polymer rheology (**a**) 1000 ppm (**b**) 1500 ppm (**c**) 2000 ppm and (**d**) 2500 ppm.

At an ambient temperature of 25 °C, Figure 5 compares the rheology of pure polymer solutions at various concentrations (1000, 1500, 2000, and 2500 ppm) with that of nanofluid-assisted polymer solutions. The results indicate that adding 0.1 wt% of silica nanoparticles to a modified synthetic polymer increased the viscosity of 1000, 1500, and 2000 ppm polymer solutions. However, at a polymer concentration of 2500 ppm, the rheology experiments showed nearly the same viscosity values for both polymer and nanofluid/polymer solutions. These findings suggested that a polymer concentration of 2000 ppm is optimum when combined with 0.1 wt% nanoparticles and increasing polymer concentration had no significant effect on viscosity. The carboxyl group in HPAM polymer and silica nanoparticles both have negative charges which are responsible to increase the polymer’s viscosity. The negative charges on the silica nanoparticles and carboxyl group repel each other when they are combined, stretching the polymer chains and enhancing the viscosity of the solution. The rheological results of pure polymer and nanofluid-assisted polymer solutions at various concentrations are shown in Table 8 and Table 9, respectively.

**Figure 6:**
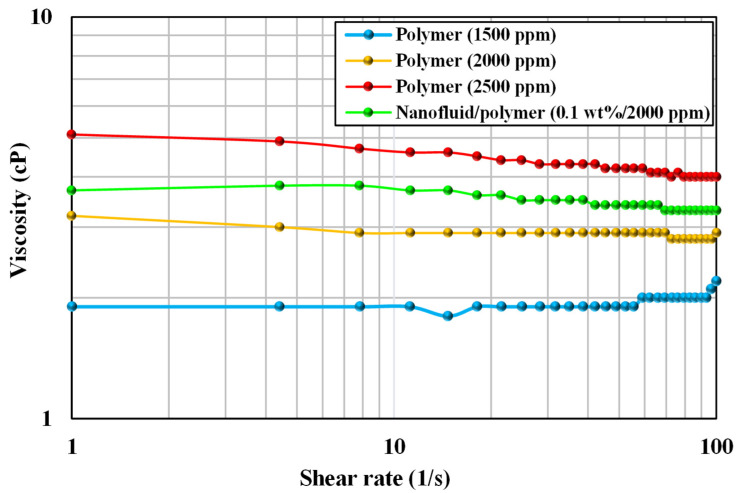
Rheology of polymers (1500, 2000, 2500 ppm) and nano-assisted polymer at 80 °C.

Figure 6 presents the rheology measurements of SAV 10 polymer at 80 °C with concentrations of 1500, 2000, and 2500 ppm. The target polymer viscosity was achieved at 2000 ppm at 80 °C, therefore, this concentration was selected for further research. The rheology results of pure polymer solutions at a high temperature of 80 °C are shown in Table 10.

As shown in Figure 6, the viscosity was increased by the combination of 0.1 wt% silica nanoparticles and the modified synthetic polymer at the selected concentration of 2000 ppm. Pure polymer (2000 ppm) and nano-polymer solutions (0.1 wt% + 2000 ppm) had viscosities of 2.9 and 3.7 cP at a shear rate of 10 1/s, respectively. Hence, silica nanoparticles increased the viscosity of the polymer by 27.6%, which might lead to a better mobility ratio. Hence, for oil displacement, the nanofluid-assisted polymer solution containing 0.1 wt% silica nanoparticles and 2000 ppm polymer was the best selection.

Furthermore, the long-term thermal stability of nanofluid-assisted polymer was also assessed in our previous study [43]. It has been observed that as hydrolysis progresses at high temperatures, the number of carboxyl groups in synthetic polymers increases. As a result, the stability of the synthetic polymer decreases at high temperatures. On the other hand, the stability of nanofluids decreases with time and at high temperatures as a result of the compression of the electric double layer, which causes nanoparticles to aggregate. The thermal stability of the designed nanofluid-assisted polymer solution was examined at 80 °C for one month. The viscosity of the solution initially decreased during the first week, but it remained stable and above 4 cp for the rest of the time. Additionally, the stability test revealed that the nanofluid-assisted polymer solution maintained 50% of its initial viscosity throughout the test period.

### 3.3. Core Flooding Experiments

**Figure 7:**
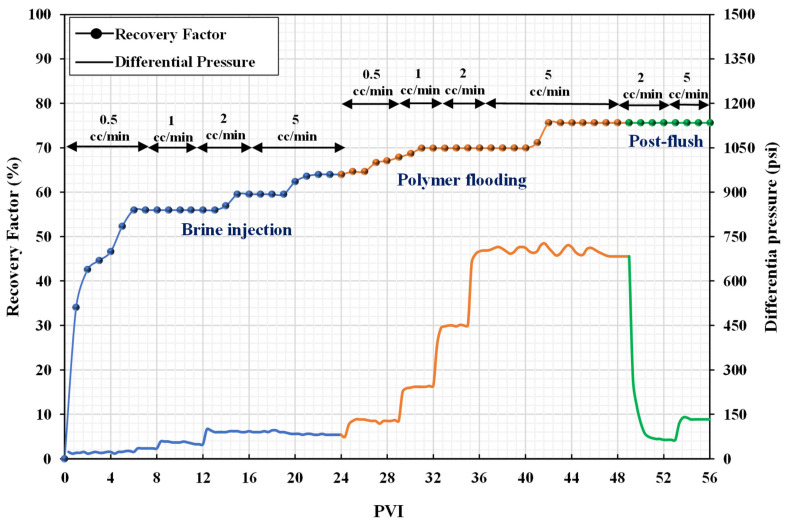
Recovery factors and differential pressure versus PVI (coreflood-1).

Considering the injection sequence for coreflood-1 from Table 6, initially, the brine of 40,000 ppm was injected into the oil-saturated core sample to recover maximum oil by waterflooding. The injection was started with 0.5 cc/min and the flow rate gradually increased in steps to a high value of 5 cc/min to mitigate capillary end effects. The flow rate was increased to the next value only when there was no oil production at the outlet. The oil recovery was stopped after 24 PV of brine injection, and the recovery factor by waterflooding was calculated to be 63.96%. Next, the polymer solution of 2000 ppm was injected to evaluate the effectiveness of polymer flooding. The additional oil recovery by polymer flooding was 11.63%. Finally, after recovering maximum oil by polymer flooding, the brine was again injected as a post-flush treatment to assess any reduction in permeability. Figure 7 shows the recovery factor and differential pressure against each pore volume injected (PVI).

Figure 7 indicates that the differential pressure increased while injecting high-viscosity polymer solution, which helped to mobilize some of the residual oil in bigger pores, even though the initial injection rate was the same as during waterflooding. The differential pressure during polymer flooding increased as the flow rate increased, reaching a maximum of 715 psi at a flow rate of 5 cc/min. To permeate larger pores and mobilize trapped oil, a high-pressure gradient is required. A measurement of polymer solution mobility reduction relative to brine was performed considering the resistance factor (RF). Furthermore, the residual resistance factor (RRF) represents the decrease in permeability caused by the polymer retention and can be estimated after a post-flush with brine. Equations (1) and (2) are used to quantify the RF and RRF, respectively.

(1) $$\mathrm{RF}=\frac{∆p_{p}}{∆{p_{w}}^{before\;polymer\;flooding}}$$

(2) $$\mathrm{RRF}=\frac{∆{p_{w}}^{after\;polymer\;flooding}}{∆{p_{w}}^{before\;polymer\;flooding}}$$

The estimated RF and RRF values were 7.34 and 1.43, respectively. This indicates that there was no decrease in permeability following polymer flooding and that the polymer solution had a high viscosity throughout the experiment.

**Figure 8:**
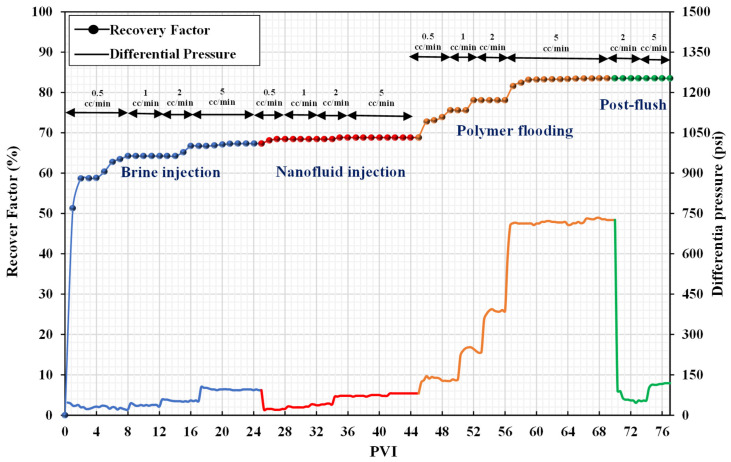
Recovery factors and differential pressure versus PVI (coreflood-2).

Recovery factor and differential pressure measurements against PV injected for coreflood-2 are shown in Figure 8. Similar to the previous test, the brine was injected at flow rates between 0.5 cc/min and 5 cc/min, and after 23 PVI, there was no oil recovery. The initial oil recovery by waterflooding was estimated to be 67.35%. In this experiment, standalone nanofluid injection (NF) and polymer flooding were used as tertiary recovery techniques. In this scenario, 0.1 wt% silica nanofluid and 2000 ppm polymer solution were used. The results indicate that silica nanoparticles reduced the capillary force, allowing the polymer solution to invade unswept pores and improve oil recovery. The nanofluid injection only increased oil recovery by 1.47%, but polymer flooding increased it by 14.71%. Compared to coreflood-1, polymer flooding recovered more oil which was due to the influence of nanoparticles on wettability alteration and capillary force reduction [48]. Therefore, more oil was displaced as a result of the same viscous force. Similar to coreflood-1, RF and RRF were calculated to be 7.56 and 1.24, respectively.

**Figure 9:**
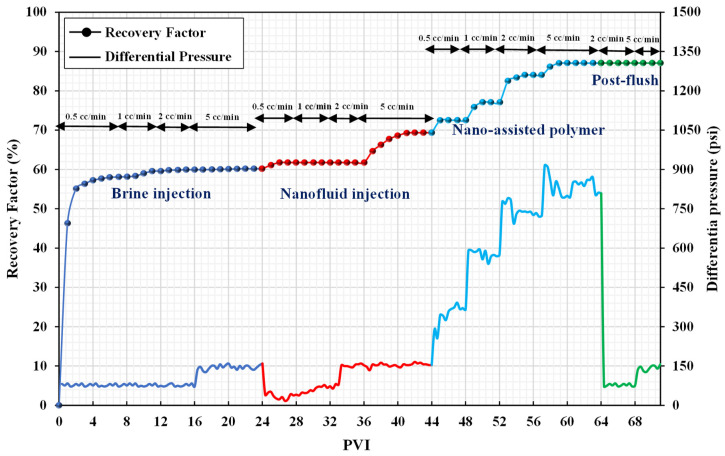
Recovery factors and differential pressure versus PVI (coreflood-3).

A similar scenario was used for brine injection during coreflood-3. Oil production stopped after 23 PV of injected brine, with an estimated recovery factor of 60% by waterflooding. After injecting 0.1 wt% nanofluid, an additional 9.15% oil recovery was obtained due to the wettability alteration mechanism. Afterward, nanofluid-assisted polymer (0.1 wt% + 2000 ppm) was injected into the core, which significantly increased the oil recovery factor to 17.7%. It was inferred that silica nanoparticles influenced the microscopic sweep efficiency, while nanofluid-assisted polymer flooding improved both the microscopic and macroscopic sweep efficiencies resulting in a higher recovery factor. At different flow rates, the differential pressures for brine and nanofluid were almost the same, reaching a maximum value of 160 psi. The presence of silica nanoparticles boosts the polymer viscosity, as was described in rheology tests. As a result, the differential pressure of the nanofluid-assisted polymer was greater than the differential pressure of the pure polymer and reached 916 psi at higher flow rates, as shown in Figure 9.

After post-flush, the RF and RFF were calculated to be 6.83 and 1.04, respectively. The RF value suggests that there was a significant improvement in the viscosity of the nanofluid-assisted polymer in the porous media while there was no decrease in permeability, as indicated by the RRF value. As a result, adding silica nanoparticles to a polymer solution reduces polymer retention in carbonate rock. This is an advantage of using a hybrid technique, which addresses a significant limitation of standalone polymer flooding.

**Figure 10:**
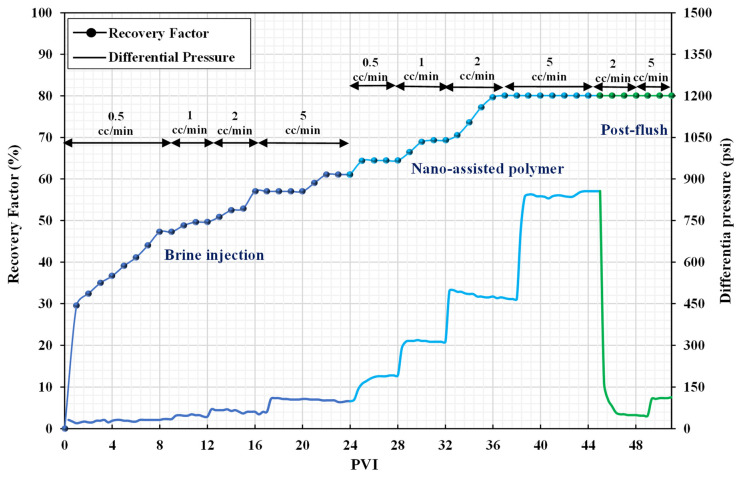
Recovery factors and differential pressure versus PVI (coreflood-4).

During coreflood-4, brine injection was similar to earlier experiments, with a recovery factor of 61.05% by waterflooding. The nanofluid-assisted polymer solution was injected immediately after waterflooding, and an additional 19.01% oil recovery was obtained. Because of the interaction between nanoparticles and rock, oil recovery increased significantly once the chemical injection process began. The presence of silica nanoparticles in the chemical slug altered the wettability of the system, thus accelerating oil recovery. High viscosity nanofluid-assisted polymer solution also improved sweep efficiencies making it possible to push more oil toward the outlet. Due to high viscosity, the differential pressure of the nano-assisted polymer was higher than that of the pure polymer and reached 856 psi at higher flow rates, as shown in Figure 10.

After post-flush, the RF was calculated to be 7.6, showing that the solution’s viscosity has improved further compared to coreflood-3. Additionally, the RRF of 1.02 indicates no permeability reduction. The key advantage of coreflood-4 is that a high oil recovery has been achieved in comparison to earlier flooding experiments in a shorter time. Moreover, there is no polymer retention in porous media, the sufficiently high viscosity of nano-assisted polymer to displace oil, and no reduction in permeability as demonstrated by the RRF value.

The relative contribution of wettability alteration by nanofluid and polymer’s viscosity improvement in the presence of nanoparticles in additional oil recovery are presented in Table 11. In corefloods-2, nanofluid altered the wettability and polymer injection recovered more oil compared to coreflood-1. Considering coreflood-1 as the base case, the relative contribution of wettability alteration in coreflood-2 in additional oil recovery was 28%. Similarly, in coreflood-3, 39% of additional oil recovery was due to wettability alteration, 18% from viscosity improvement and 43% was due to mobility control and viscous forces. In the last coreflood, wettability alteration contributed to 8% additional oil recovery, 31% from viscosity improvement and 61% was due to mobility control and viscous forces.

### 3.4. Selection of the Optimum Oil Displacement Scenario

**Figure 11:**
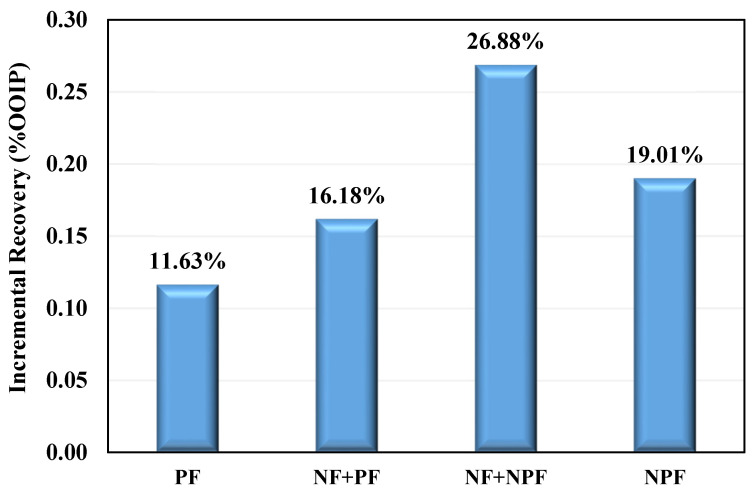
Comparison of incremental oil recovery for each scenario.

Figure 11 shows a relative comparison of oil recovery in all coreflood experiments using 40,000 ppm brine, 0.1 wt% silica nanofluid, the polymer of 2000 ppm concentration, and nanofluid-assisted polymer solution (0.1 wt% silica nanoparticles + 2000 ppm polymer) at 80 °C. The highest oil recovery was achieved in coreflood-3 making it an optimum and suitable hybrid EOR technique for maximizing oil recovery from carbonate reservoirs. Silica nanoparticles reduce capillary forces, increasing oil recovery while injecting nanofluid-assisted polymer solution improved both microscopic and macroscopic sweep efficiency. The RF estimation showed that the combination of modified synthetic polymer and silica nanoparticles increased the viscosity of the injected fluid in porous media, resulting in better mobility control. Moreover, silica nanoparticles prevented polymer retention in porous media, as explained by RRF calculations. Thus, the synergy of different mechanisms such as wettability alteration, mobility control, and change of disjoining pressure all led to improved oil recovery. Therefore, these mechanisms considerably increase the recovery factor, making the third scenario the optimum choice for the EOR strategy.

## 4. Conclusions

The demand for crude oil and its byproducts is widespread, hence it is essential to maximize oil production from hydrocarbon reservoirs. In this research, polymer flooding, nanofluid flooding, and hybrid flooding methods were investigated. The synergy of nanoparticles and polymer played a vital role in recovering maximum oil from the core. The key advantages of the hybrid approach are:

- Rock wettability can be changed using silica nanofluids. The wettability of carbonate rock was most effectively changed from an oil-wet to a water-wet with a deviation of 45.6° state at 0.1 wt% nanoparticles, making it the optimum concentration for oil displacement.
- Rheology experiments proved that adding silica nanoparticles to a polymer solution increased the fluid viscosity by 27.6% at 80 °C.
- Contact angle measurements and rheological experiment results showed that a nanofluid-assisted polymer solution containing 0.1 wt% silica nanoparticles and 2000 ppm polymer was the best selection.
- As an effective EOR technique, injecting silica nanofluid followed by a nano-assisted polymer resulted in a maximum incremental oil recovery of 26.88% in the third scenario.
- Corresponding RF and RRF values of 6.83 and 1.04 the addition of silica nanoparticles to the polymer prevents polymer retention, therefore, injection fluid viscosity increased in porous media with no permeability reduction; Thus, the combination of silica nanoparticles and the polymer is more effective than pure polymer solution due to synergy of different mechanisms.

## Figures and Tables

**Table 1:** Dimensions of the core samples.

Core No.	Length (cm)	Dry Weight (g)	V_p_ (mL)	V_b_ (mL)	ϕ (%)
1	7.95	200.28	17.09	90.68	18.85
2	7.27	182.04	16.13	82.93	19.45
3	7.13	180.09	15.09	81.33	18.56
4	7.15	178.44	15.36	81.56	18.84

**Table 2:** Permeability and saturation results.

Core No.	S_oi_ (%)	S_wi_ (%)	k_abs_ (mD)	k_eff-oil_ (mD)
1	72.3	27.7	30.27	14.92
2	79.9	20.1	39.23	23.92
3	79.5	20.5	43.43	24.06
4	80.25	19.75	25.03	15.25

**Table 3:** Crude oil properties.

Temperature (°C)	Dynamic Viscosity (cp)	Density (g/cm^3^)
25	5.66	0.84
80	2.89	0.81

**Table 4:** Ionic composition of brines.

Ions	Formation Water (ppm)	Injection Brine (ppm)
Na^+^ and K^+^	81,600	13,600
Ca^2+^	1470	1590
Mg^2+^	9540	245
Cl^−^	90,370	15,062
TDS	182,980	40,000

**Table 5:** Nanoparticle characteristics.

Nanoparticle	Size (nm)	Specific Surface Area (m^2^/g)	Morphology	Density (g/cm^3^)	Purity
Silicon dioxide (SiO_2_)	10–20	640	Spherical	2.4	99.50%

**Table 6:** Injection sequence for each core flooding.

Coreflood No.	Injection Sequence
1	Brine → Polymer → Post-flush
2	Brine → Nanofluid → Polymer → Post-flush
3	Brine → Nanofluid → Nanofluid-polymer → Post-flush
4	Brine → Nanofluid-polymer → Post-flush

**Table 7:** Measured zeta potentials.

Silica Nanofluid (wt%)	Zeta Potential (no Salt), mV	Zeta Potential (with Salt), mV
0.05	−39.7	−4.06
0.1	−42.5	−6.3
0.15	−39.5	−3.12

**Table 8:** Rheology of pure polymer solutions.

Solution	Temperature	Shear Rate, 1/s	Polymer Concentration, ppm	Viscosity, cP
Polymer	25	10	1000	2.5
			1500	5
			2000	5.75
			2500	8.7

**Table 9:** Rheology of nanofluid/polymer solutions.

Solution	Temperature, °C	Shear Rate, 1/s	SiO_2_ Concentration, wt%	Polymer Concentration, ppm	Viscosity, cP
Nano-assisted polymer	25	10	0.1	1000	3.32
				1500	6.3
				2000	6.15
				2500	8.63

**Table 10:** Rheology of pure polymer solutions at high temperatures.

Solution	Temperature, °C	Shear Rate, 1/s	Polymer Concentration, ppm	Viscosity, cP
Polymer	80	10	1500	1.9
			2000	3.1
			2500	4.6

**Table 11:** The relative contribution of wettability alteration and polymer’s viscosity improvement.

Sr. No.	Injection Fluid	Incremental Recovery (%)	Recovery Mechanism	Total Incremental Recovery (%)
coreflood-1	polymer	11.63	mobility control + viscous forces	11.63
coreflood-2	nanofluid	1.47	wettability alteration	16.18
	polymer	14.71	mobility control + viscous forces	
coreflood-3	nanofluid	9.15	wettability alteration	26.88
	nanofluid-assisted polymer	17.7	better performance of the polymer in the presence of nanofluid	
coreflood-4	nanofluid-assisted polymer	19.01	wettability alteration + better performance of the polymer in the presence of nanofluid	19.01

## Data Availability

Not applicable.
